# Material Synthesis and Device Aspects of Monolayer Tungsten Diselenide

**DOI:** 10.1038/s41598-018-23501-4

**Published:** 2018-03-27

**Authors:** Zihan Yao, Jialun Liu, Kai Xu, Edmond K. C. Chow, Wenjuan Zhu

**Affiliations:** University of Illinois at Urbana_Champaign, Urbana, United States

## Abstract

In this paper, we investigate the synthesis of WSe_2_ by chemical vapor deposition and study the current transport and device scaling of monolayer WSe_2_. We found that the device characteristics of the back-gated WSe_2_ transistors with thick oxides are very sensitive to the applied drain bias, especially for transistors in the sub-micrometer regime. The threshold voltage, subthreshold swing, and extracted field-effect mobility vary with the applied drain bias. The output characteristics in the long-channel transistors show ohmic-like behavior, while that in the short-channel transistors show Schottky-like behavior. Our investigation reveals that these phenomena are caused by the drain-induced barrier lowering (short-channel effect). For back-gated WSe_2_ transistors with 280 nm oxide, the short-channel effect appears when the channel length is shorter than 0.4 µm. This extremely long electrostatic scaling length is due to the thick back-gate oxides. In addition, we also found that the hydrogen flow rate and the amount of WO_3_ precursor play an important role in the morphology of the WSe_2_. The hole mobility of the monolayer WSe_2_ is limited by Columbic scattering below 250 K, while it is limited by phonon scattering above 250 K. These findings are very important for the synthesis of WSe_2_ and accurate characterization of the electronic devices based on 2D materials.

## Introduction

WSe_2_ is an important member of the transition metal dichalcogenide (TMDC) family due to its smaller effective electron and hole masses compared to most of the other TMDCs^1^, and more importantly due to its ambipolar characteristics^2^. The small effective mass implies high carrier mobilities. The hole mobility of WSe_2_ is reported to reach 500 cm^2^/V-s at room temperature and 2.1 × 10^3^ cm^2^/V-s at 5 K^3,4^. The ambipolar conduction is essential for complementary metal-oxide semiconductor (CMOS) circuits such as inverters, since most of the TMDCs (such as MoS_2_, MoSe_2_, and WS_2_), are naturally n-type doped. Although MoTe_2_ and black phosphorus have also been reported to show p-type conduction^5–8^, these materials are less stable in ambient conditions. Electrical properties of WSe_2_ including quantum oscillations, carrier mobilities, contacts, and polarity controls, have been studied and various electronic devices based on WSe_2,_ including metal-oxide field-effect transistors (MOSFETs), tunneling devices, bipolar transistors, and integrated circuits, have been demonstrated^2,3,9–22^. However, most of these studies focus on exfoliated WSe_2_ flakes. For practical applications, synthesis of large area WSe_2_ with controllable layer thickness and quality is essential. There are several recent reports on the growth of thin WSe_2_ using chemical vapor deposition (CVD)^23–26^, and metal-organic CVD (MOCVD)^27,28^. However, study of the electrical transport of CVD grown WSe_2_ is still lacking. In this paper, we systematically investigate the growth of monolayer WSe_2_ by CVD and study the current transport of CVD WSe_2_, including short-channel effect, temperature dependence of carrier mobility, subthreshold swing, and interface states.

## Results and Discussion

### Synthesis of CVD WSe_2_

**Figure 1:**
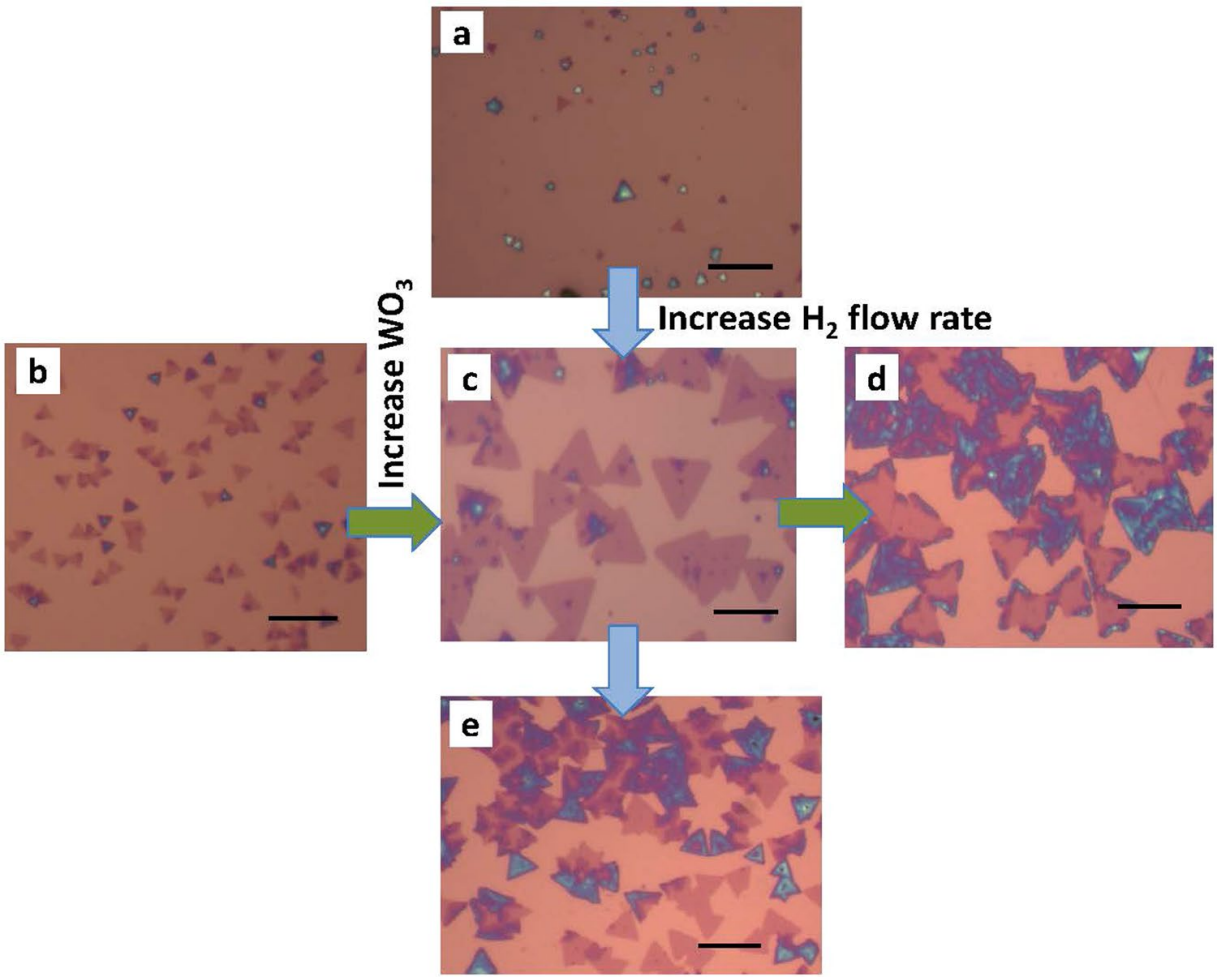
Optical image of CVD WSe_2_ grown at various hydrogen flow rates and WO_3_ precursor amounts: (**a**) H_2_ flow rate 15 sccm, WO_3_ quantity 150 mg; (**b**) H_2_ flow rate 20 sccm, WO_3_ quantity 100 mg; (**c**) H_2_ flow rate 20 sccm, WO_3_ quantity 150 mg; (**d**) H_2_ flow rate 20 sccm, WO_3_ quantity 200 mg; (**e**) H_2_ flow rate 25 sccm, WO_3_ quantity 150 mg. The scale bars in the images are 10 µm.

Solid precursor WO_3_ and Se power were used to synthesize WSe_2_ on Si/SiO_2_ substrate in a CVD chamber. Various growth conditions have been investigated, including hydrogen flow rate, WO_3_ precursor amount, growth temperature, and argon flow rate. We found that these growth parameters can significantly influence the morphology of the WSe_2_. Figure 1a–e show the optical images of the synthesized WSe_2_ with various hydrogen flow rates and WO_3_ precursor amounts. For a given flow rate of argon carrier gas [60 standard cubic centimeters per minute (sccm)], when the hydrogen flow rate increases from 15 sccm to 20 sccm, the grain size of WSe_2_ increases dramatically. However, when the hydrogen flow rate further increases to 25 sccm, the grain size decreases and multilayer stacks start to form. This is because selenium has very low chemical reactivity. A strong reducer such as hydrogen is needed in the selenization reduction of WO_3_. However, if the hydrogen flow rate is too high, the reduction of WO_3_ to W happens very quickly and the WO_3_ powder only lasts for a very short time, which results in small grains of thick WSe_2_. The amount of the precursors loaded in the chamber also plays an important role. For a given amount of Se precursor [(300 milligram (mg)], increasing the quantity of WO_3_ precursor from 100 mg to 150 mg can effectively increase the grain size. However, further increase of WO_3_ to 200 mg will result in the growth of multi-layer WSe_2_.

**Figure 2:**
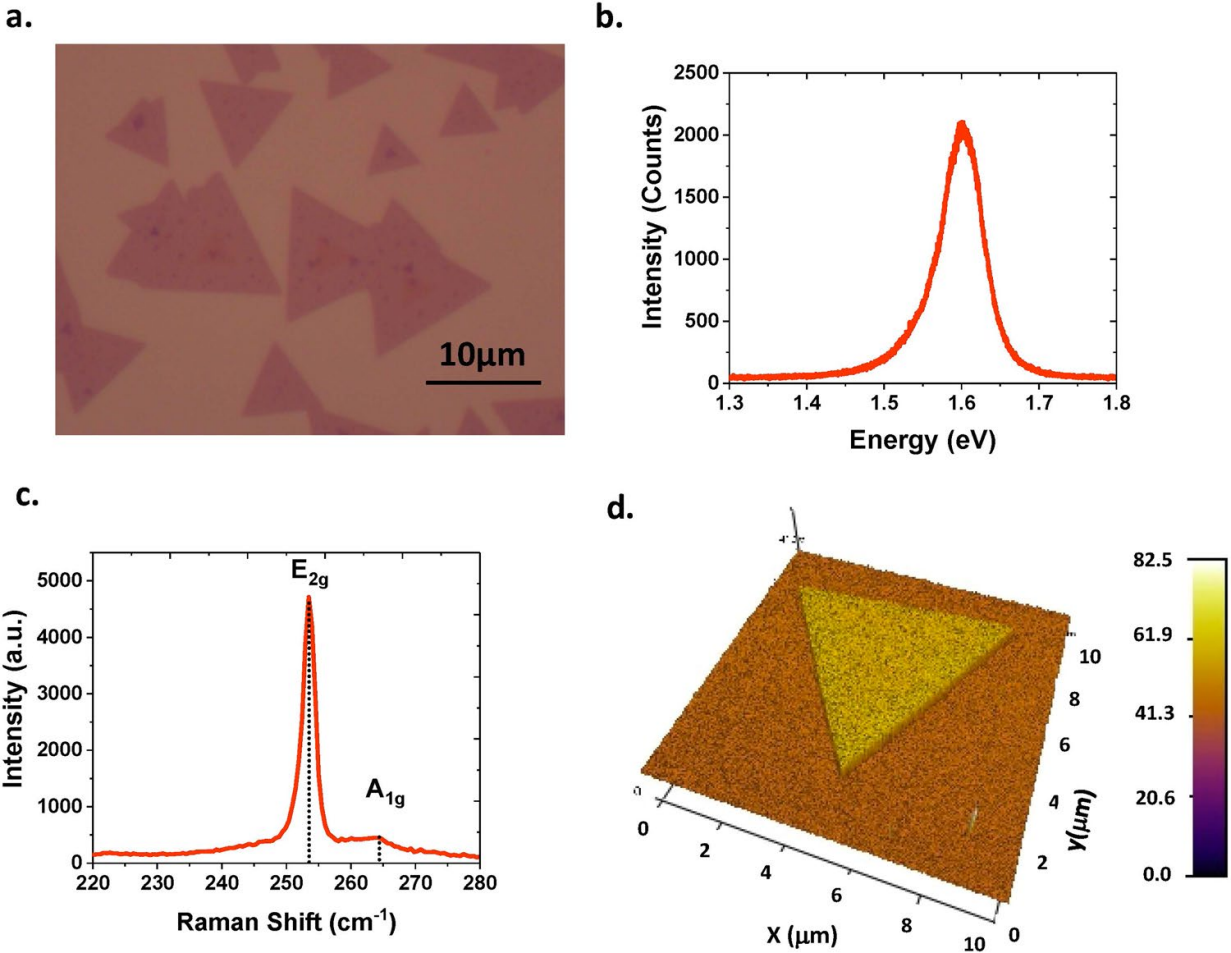
Microscopic characterization of monolayer WSe_2_. (**a**) Optical image, (**b**) Photoluminescence spectrum, (3) Raman spectrum, and (4) AFM phase image.

The optimized growth condition for WSe_2_ using this CVD system is 150 mg of WO_3_ and 300 mg of Se powder, ambient pressure, 875 °C growth temperature, 70/20 sccm flow of Ar/H_2_, and 10~15 minutes growth duration. Perylene-3,4,9,10-tetracarboxylic acid tetra potassium (PTAS) was used as the seeding promoter. With the optimized growth condition, high-quality monolayer WSe_2_ was obtained. Figure 2a–d shows the typical optical image, photoluminescence (PL) spectrum, Raman spectrum, and atomic force microscopy (AFM) phase image of the synthesized WSe_2_. Bright light emission at ∼1.60 eV and symmetric single PL peak suggest the direct band gap nature of monolayer WSe_2_, showing good agreement with other recent reports about PL of monolayer WSe_2_^24–26,29–32^. The E_2g_ and A_1g_ modes in the Raman spectrum are at 253.5 cm^−1^ and 264.2 cm^−1^, respectively. Comparing the peak position with the spectrum obtained from the exfoliated monolayer WSe_2_^33^, we verify that the WSe_2_ film is monolayer. The AFM phase image shows a clear triangle pattern. From the AFM step height profile, the thickness of the WSe_2_ is measured as ~0.54 nm, confirming its monolayer character.

### Device scaling of the back-gated WSe_2_ transistors

**Figure 3:**
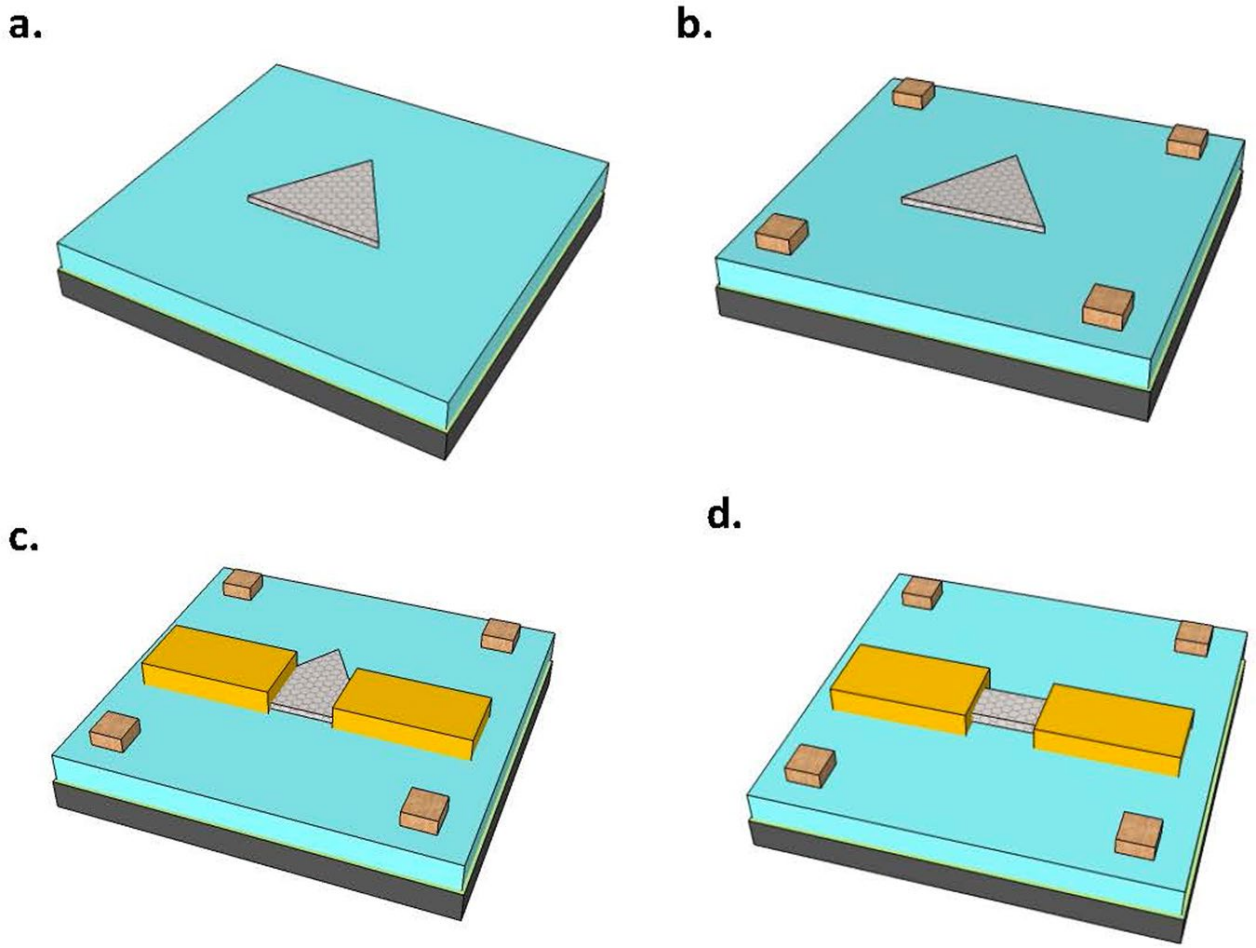
Illustration of the fabrication of the WSe_2_ devices. (**a**) Synthesize WSe_2_ on SiO_2_/Si substrate using CVD. (**b**) Form alignment marks using ebeam lithography, metal deposition and lift-off. (**c**) Form source/drain electrodes using ebeam lithography, metal deposition and lift-off. (**d**) Define channel by ebeam lithography and oxygen plasma etching.

WSe_2_ transistors with various gate lengths (from 0.1 µm to 5 µm) are fabricated using Pd as metal contacts. Figure 3 illustrated the process flow of the WSe_2_ devices. The alignment marks with Ti/Au metals were formed by ebeam lithography, metal deposition and lift-off. Hall-bars and transistors were designed for individual WSe_2_ triangles. The source/drain electrodes (40 nm Pd) were formed by ebeam lithography, metal evaporation and lift-off. The WSe_2_ channel is defined by ebeam lithography and oxygen plasma etching. The electrical characteristics were measured in vacuum at various temperatures.

**Figure 4:**
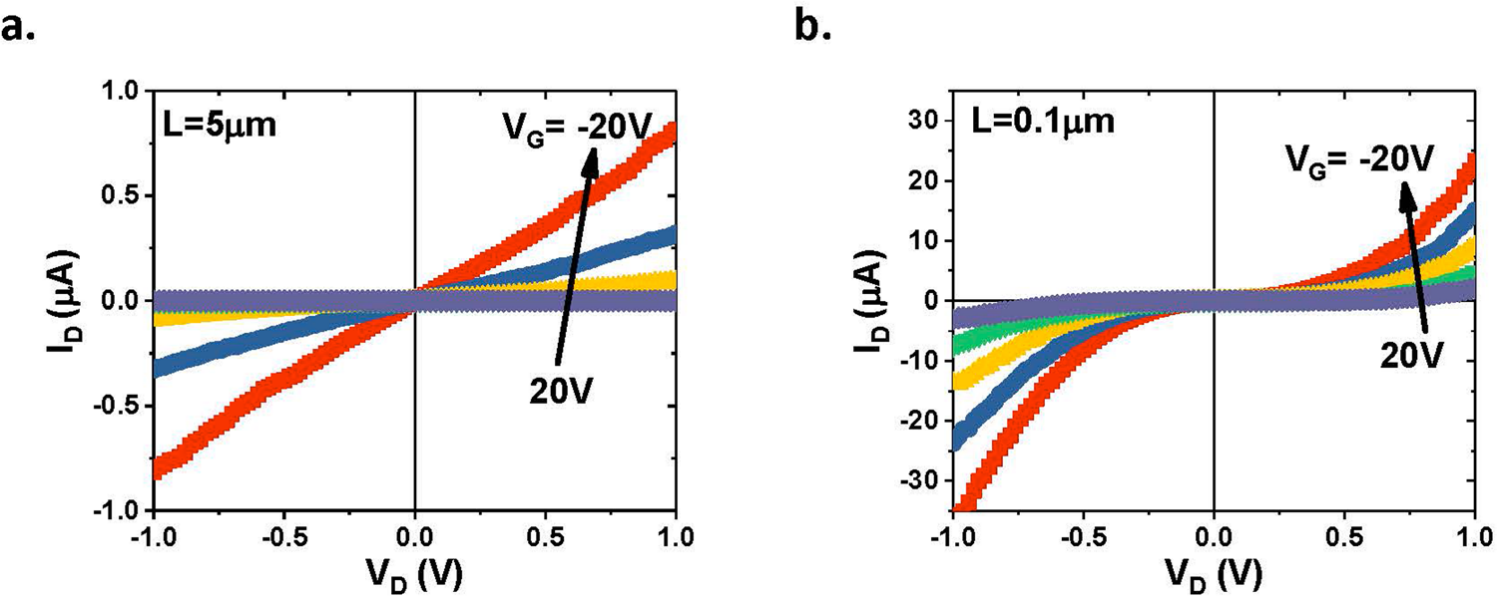
The output characteristics of the CVD WSe_2_ transistors with (**a)** channel length L = 5 µm, and (**b**) channel length L=0.1 µm.

Surprisingly, in long-channel transitions (L = 5 µm), the output characteristics (I_D_~V_D_ curves) are linear, indicating ohmic contacts, shown in Fig. 4a, while in short-channel transistors (L = 0.1 µm), the I_D_~V_D_ curves are non-linear, shown in Fig. 4b, resembling Schottky contact characteristics. Since both the long- and short-channel transistors are fabricated on the same wafer with nearly identical WSe_2_ and Pd metals. It is very unlikely that they formed different types of contacts. Our further investigation reveals that these non-linear output characteristics in the WSe_2_ transistors are not related to the metal contacts, but due to the short-channel effect, which will be discussed below.

**Figure 5:**
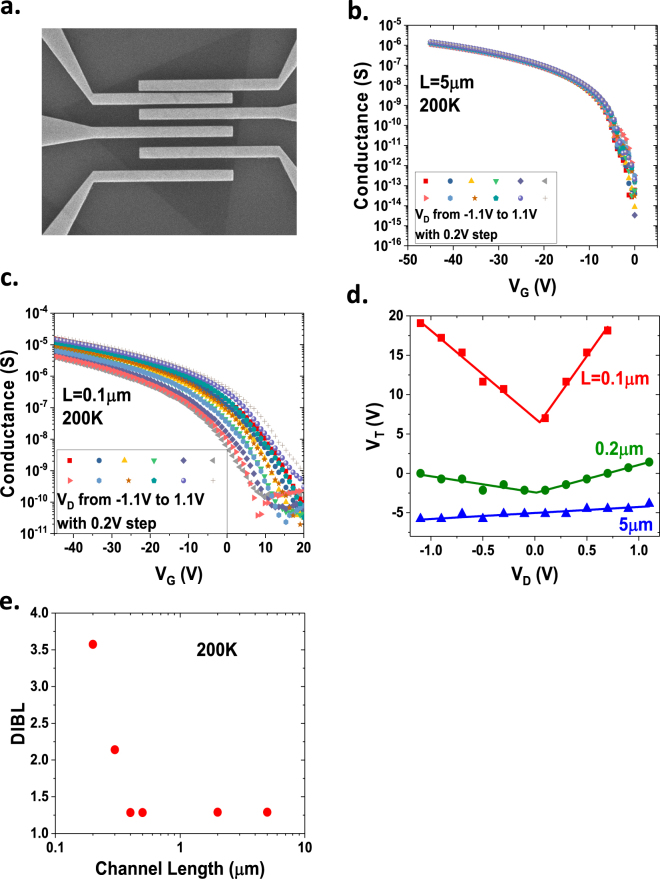
Short-channel effect in WSe_2_ transistors. (**a**) SEM image of a WSe_2_ TLM structure consisting of five transistors with various channel lengths. (**b**) Conductance as a function of gate voltage in a long-channel transistor (L = 5 µm) measured at various drain voltages. (**c**) Conductance as a function of gate voltage in a short-channel transistor (L = 0.1 µm) measured at various drain voltages. (**d**) Threshold voltage as a function of drain voltage for WSe_2_ transistors with various channel lengths from 0.1 µm to 5 µm. (**e**) Drain-induced barrier lowering (DIBL) of the WSe_2_ transistors as a function of channel length.

The transfer characteristics (I_D_~V_G_ curves) were measured at various drain voltages for WSe_2_ transistors with various gate lengths in a transmission line micro (TLM). A typical SEM image of the TLM structure is shown in Fig. 5a. (Note, in order to avoid the SEM damage on the monolayer WSe_2_ in the electronic device, the SEM is taken on a neighboring device with same layout on the same wafer as the device tested electrically). The channel conductances at various drain voltages for a long-channel (L = 5 µm) and a short-channel (L = 0.1 µm) transistor, are shown in Fig. 5b and c. For the long-channel transistor, the conductance is nearly independent of the drain voltages. For the short-channel transistor, however, there is a large dispersion of the conductance, especially at the subthreshold regime. Figure 5d plots the threshold voltage as a function of drain voltage for transistors with channel lengths of *L* = 0.1 *μ*m, 0.2 *μ*m, and 5 *μ*m. We can see that the threshold voltage increases dramatically as the amplitude of the drain voltage, |V_D_|, increases for the short channels (*L* = 0.1 *μ*m and 0.2 *μ*m), while it is nearly constant for the long channel (*L* = 5 *μ*m). This positive shift of the threshold voltage at high drain bias in these short-channel p-type transistors is a result of the fact that the energy barrier between the source and drain is lowered by the high drain voltage and thus the transistors are much easier to turn on. The drain-induced barrier lowering (DIBL) can be estimated using the equation ${\rm{DIBL}}=\frac{{V}_{t}^{DD}-{V}_{t}^{Low}}{{V}_{DD}-{V}_{D}^{Low}}$, where ${V}_{t}^{DD}\,$is the threshold voltage measured at a supply voltage (high drain voltage), ${V}_{t}^{Low}\,$is the threshold voltage measured at a very low drain voltage, *V*_*DD*_ is the supply voltage (high drain voltage), ${V}_{D}^{Low}$ is the low drain voltage. Here we use drain biases of 0.1 V and 1.1 V to extract the DIBL. Figure 5e shows the DIBL of the back-gated WSe_2_ transistor as a function of the channel length. The upturn of the DIBL does not show up until the channel length is shorter than 0.4 µm. Since the back-gate oxide is very thick (280 nm in this case), the electrostatic scaling length is very long. The electrostatic scaling length can be estimated by the equation $\lambda =\sqrt{{t}_{ox}{t}_{s}{\varepsilon }_{s}/{\varepsilon }_{ox}}$, where *t*_*ox*_ is the oxide thickness, *t*_*s*_ is the semiconductor thickness, *ε*_*s*_ is the semiconductor dielectric constant, and *ε*_*ox*_ is the oxide dielectric constant^34,35^. For the back-gated WSe_2_ transistors, assuming *t*_*ox*_ = 280 nm, *t*_*s*_ ≈ 0.54 nm, *ϵ*_*s*_ ≈ 7, and *ϵ*_*ox*_ ≈ 3.9, respectively, the electrostatic scaling length is estimated as *λ* ≈ 0.064 μm. For a planar device, the minimum channel length needed to preserve the long-channel behavior is typically 4–5 times the electrostatic scaling length, which corresponds to *L* = 0.26~0.32 μm in this case. This is consistent with our DIBL results, which reveal that the short-channel effect starts to show up when the channel is shorter than 0.4 μm. As the energy barrier between source and drain reduces, the drain current will increase exponentially. This explains the dramatic increase of the drain current at high drain voltages in the I_D_~V_D_ plots for the short-channel transistors, shown in Fig. 4b.

### Carrier mobility in CVD WSe_2_

**Figure 6:**
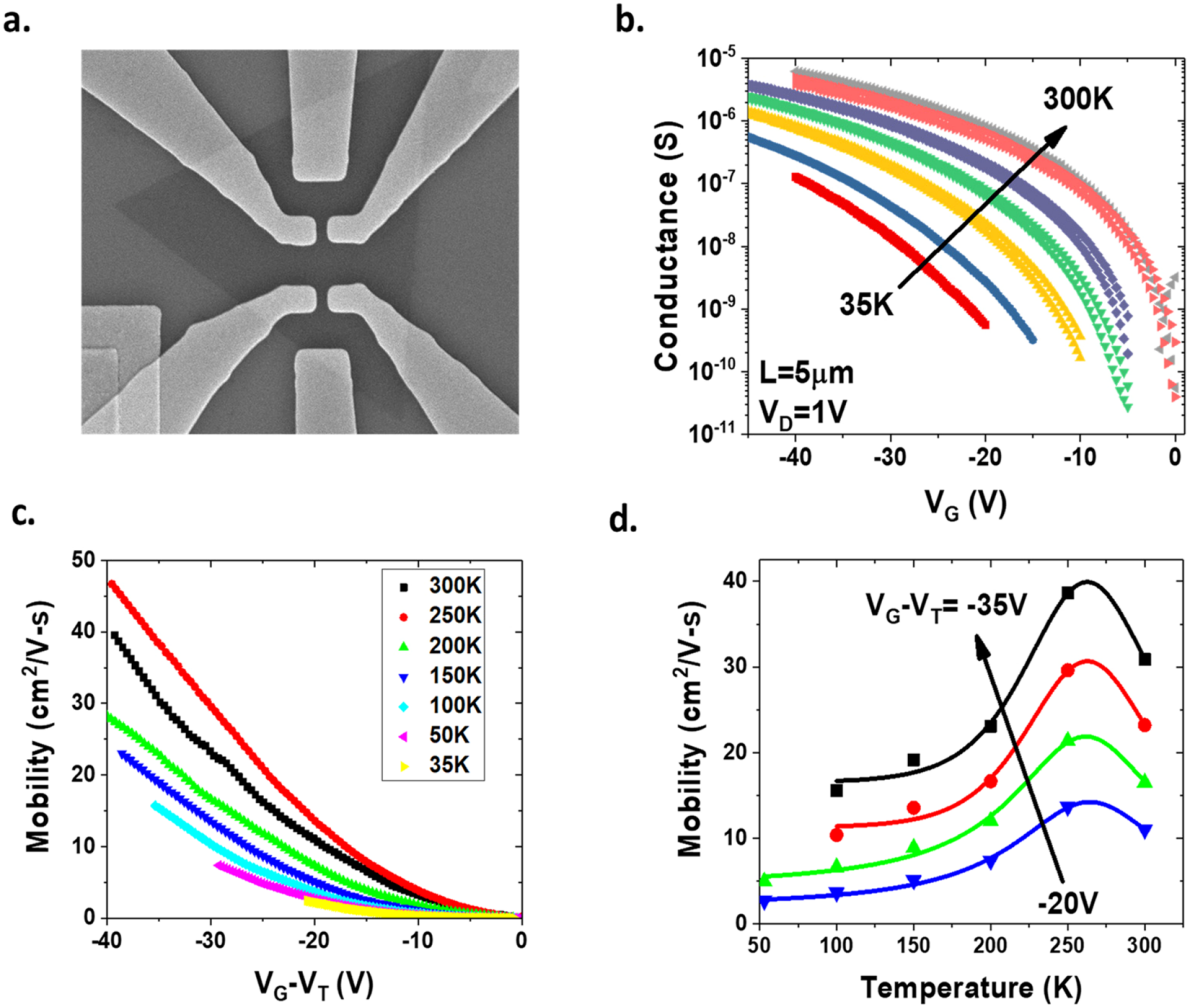
Carrier mobility of CVD WSe_2_. **(a**) SEM image of a WSe_2_ Hall-bar device. (**b**) Four-point conductance as a function of gate voltage measured at various temperatures. (**c**) Extracted field-effect mobility as a function of gate overdrive, *V*_*G*_ − *V*_*T*_, at various temperatures. (**d**) Temperature dependence of the field-effect mobility at various gate overdrives.

The carrier mobility is an important indicator of the quality of the semiconductor. The carrier mobility of the CVD WSe_2_ is studied in the long-channel transistors to eliminate the possible short-channel effects, and the conductances were measured using the four-point method to minimize the impact of the contact resistances. Figure 6a shows the SEM image of a typical Hall-bar device. The four-point conductance as a function of gate voltage measured of a WSe_2_ Hall-bar device at various temperatures is shown in Fig. 6b. Note the SEM and conductance are taken on two separate devices with the same layout on the same wafer to avoid the impact of SEM scan on the electrical performance of the device. The field-effect mobility is extracted from the four-point conductance, using the equation $\mu =\frac{1}{(W/L){C}_{ox}}\frac{\partial \sigma }{\partial {V}_{G}}$, where *σ* is the four-point conductance, *V*_*G*_ is the gate voltage, *W* is channel width, *L* is the channel length, and *C*_*ox*_ is the oxide capacitance^36^. The extracted field effect mobility as a function of gate voltage is shown in Fig. 6c. As the transistor is biased further into the inversion, the mobility increases because of the screening effect due to the inversion charge, which reduces the Coulomb scattering. The temperature dependence of the mobility shows a turn-around behavior at 250 K, shown in Fig. 6d. At low temperatures, the mobility increases with increasing temperature, and at temperatures above 250 K, the mobility decreases with increasing temperature. At low temperatures, Coulomb scattering dominates. When the temperature increases, the carrier velocity increases, which can reduce the influence of the Coulomb scattering from the charged impurities. At high temperatures, phonon scattering dominates. Increasing temperature will increase the lattice vibration and reduce the mobility. Seven Hall-bar devices were measured to determine the mobility and carrier density. At *V*_*G*_ − *V*_*T*_ = −30 *V*, i.e. carrier density of ~2.3 × 10^12^ cm^−2^, the average mobility is ~24.8 cm^2^/V-s, the maximum mobility is 46.1 cm^2^/V-s, and the minimum mobility is 7.9 cm^2^/V-s. Note, these electrical characteristics were measured after source/drain formation and before the channel is etched into rectangular shape. After the sample experienced additional process steps, including lithography, oxygen plasma etching and resist strip, and additional characterizations such as SEM, the measured mobilities of the Hall-bar devices are significantly lower, as shown in Fig. S1 in the supplementary information. One possible cause of this mobility degradation is that the additional process steps and the high-energy electron beam in SEM degraded the monolayer WSe_2_ channel. The other possible reason for the lower extracted mobility after channel-cut is that there are parasitic current path in the triangular-shaped channel before the channel-cut, which may cause overestimation of the channel mobility. The gate voltage and temperature dependence of the channel mobilities in a WSe_2_ Hall-bar devices after channel-cut and SEM scan are shown in Fig. S2. The mobilities of the WSe_2_ device after channel-cut show similar temperature and gate voltage dependence as the one before the channel-cut.

### Interface states of the WSe_2_ transistors

**Figure 7:**
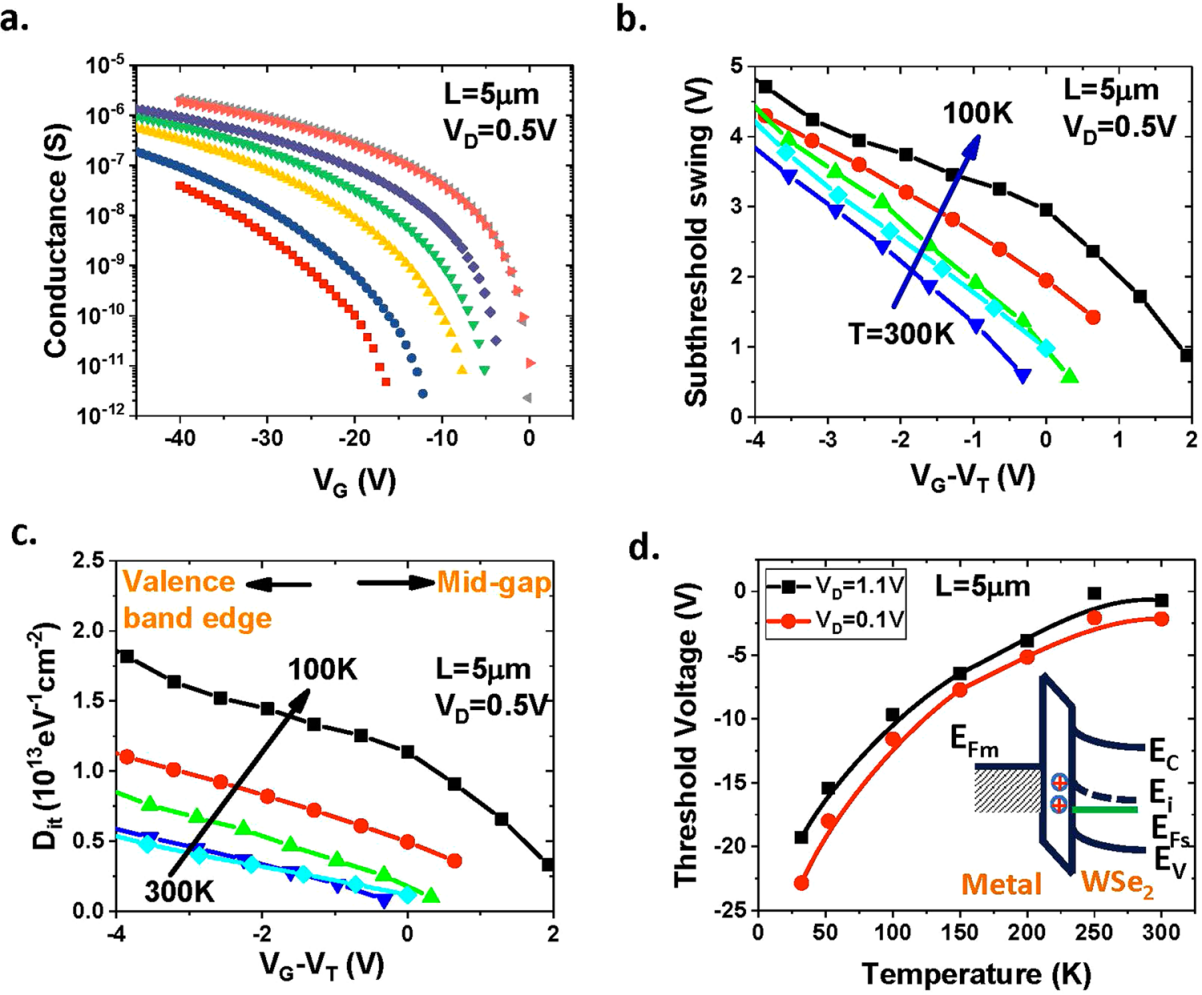
Interface states in back-gated WSe_2_ transistors. (**a**) Two-point conductance as a function of gate voltage measured at various temperatures. (**b**) Subthreshold swing as a function of gate overdrive, *V*_*G*_ − *V*_*T*_, at various temperatures. (**c**) Extracted interface trap density as a function of gate overdrive. (**d**) Temperature dependence of the threshold voltage. The inset illustrates the positive interface trapped charges located at the WSe_2_/oxide interface when the gate is biased at the threshold voltage.

The interface trap density is another key parameter for semiconductors, which directly impacts the performance of metal-oxide field-effect transistors (MOSFETs), tunneling field-effect transistors (TFETs), and tunneling diodes. The interface trap density of the CVD WSe_2_ is evaluated by measuring the subthreshold swing at various temperatures on the long-channel transistors. Figure 7a shows the I_D_~V_D_ curves measured at various temperatures for a transistor with gate length *L* = 5 *μ*m. The subthreshold swing is defined as the gate voltage required to vary the subthreshold current by one decade: $SS=\partial {V}_{G}/\partial log({I}_{D})$, where *I*_*D*_ is the drain current at the subthreshold regime. Figure 7b shows the measured subthreshold swing as a function of gate voltage. The subthreshold swing is typically a function of depletion capacitance, *C*_*D*_, and temperature. If there are interface states, an interface-state capacitance, *C*_*it*_, will be in parallel with the *C*_*D*_. Then the subthreshold swing can be modeled as $SS=\frac{kT}{q}ln(10)(1+\frac{{C}_{D}+{C}_{it}}{{C}_{ox}})$^36^. For transistors with atomically thin channel (monolayer WSe_2_), it is assumed that the device is fully depleted and *C*_*D*_ ≈ 0. Then the interface capacitance, *C*_*it*_, can be extracted. The interface trap density is related to *C*_*it*_ by the equation ${D}_{it}=\frac{{C}_{it}}{q}$, where *q* is the elementary charge. Figure 7c shows the extracted interface trap density, *D*_*it*_, as a function of gate overdrive, *V*_*G*_ − *V*_*T*_, at various temperatures. As the gate overdrive, *V*_*G*_ − *V*_*T*_, decreases, i.e. as the Fermi level at the WSe_2_/oxide interface approaches the valence band edge, the interface trap density increases, which is similar to the energy distribution of the interface traps in silicon.

The threshold voltages of these back-gated WSe_2_ transistors are also strongly dependent on the temperatures. Figure 7d shows the threshold voltage as a function of temperature. The threshold voltage increases monotonically as the temperature increases from 35 K to 300 K. This can be explained by the temperature dependence of the interface trapped charges. The interface state typically has two types: the donor-like and the acceptor-like interface states. The donor-like interface states are neutral when filled with electrons and positive when empty, whereas the acceptor-like interface states are negative when filled and neutral when empty. Assuming that the interface states below the midgap *E*_*i*_ are donor-like, while those above *E*_*i*_ are acceptor-like, similar to silicon, the interface states will be positively charged at threshold voltage in p-type transistors, where the Fermi level is below the midgap. This will result in a negative shift in the threshold voltage as compared to the one without interface states. As the temperature increases, the Fermi level is closer to the mid-gap at the threshold voltage, and the total amount of the positive trapped charges reduces, resulting in positive shift of the threshold voltages. The desorption of the moisture from the WSe_2_ surface as the samples warm up could also cause a threshold voltage shift in these back-gated transistors.

## Conclusion

In summary, we have systematically investigated the synthesis of monolayer WSe_2_ using CVD and studied the electric transport of CVD WSe_2_. We found that short-channel effect plays an important role in the back-gated WSe_2_ transistors when the channel length is in the sub-micrometer regime. The drain-induced barrier lowering can result in variations of the threshold voltages and over- or under-estimation of the carrier mobilities. This short-channel effect can also lead to misjudgment of the metal contacts, as the output characteristics of a transistor with ohmic contacts can show Schottky-like behavior. For back-gated WSe_2_ transistors with 280 nm gate oxide, the DIBL starts to show an upturn when the channel length is shorter than 0.4 µm. This extremely long electrostatic scaling length is due to the thick oxide. These findings will be very important for accurate and unified characterization and analysis of 2D electronic devices with back-gate structures. In addition, we also found that the hydrogen flow rate and the amount of WO_3_ precursor can significantly influence the morphology of the WSe_2_. Large work function metal Pd forms ohmic contact to the monolayer WSe_2_. The interface trap density of WSe_2_ was extracted from the subthreshold swings. The interface trap density of CVD WSe_2_ increases as the energy level approaches the valence band edge. These findings will enrich the knowledge of electric transport in CVD WSe_2_ and the scaled electronic devices based on monolayer TMDCs.

## Electronic supplementary material

Supplementary information
