# Supplementary material for: Material Synthesis and Device Aspects of Monolayer Tungsten Diselenide
Source: Sci Rep. 2018 Mar 27;8:5221. doi: 10.1038/s41598-018-23501-4 (PMC5869716; doi:10.1038/s41598-018-23501-4)
Supplement: Supplementary file 1 — Supplementary information [file 41598_2018_23501_MOESM1_ESM.pdf]

Supplementary Information

Material Synthesis and Device Aspects of Monolayer Tungsten Diselenide

Zihan Yao, Jialun Liu, Kai Xu, Edmond K.C. Chow, and Wenjuan Zhu

University of Illinois at Urbana-Champaign, Department of Electrical and Computer Engineering

1. Impact of channel etching and SEM scan on the carrier mobility

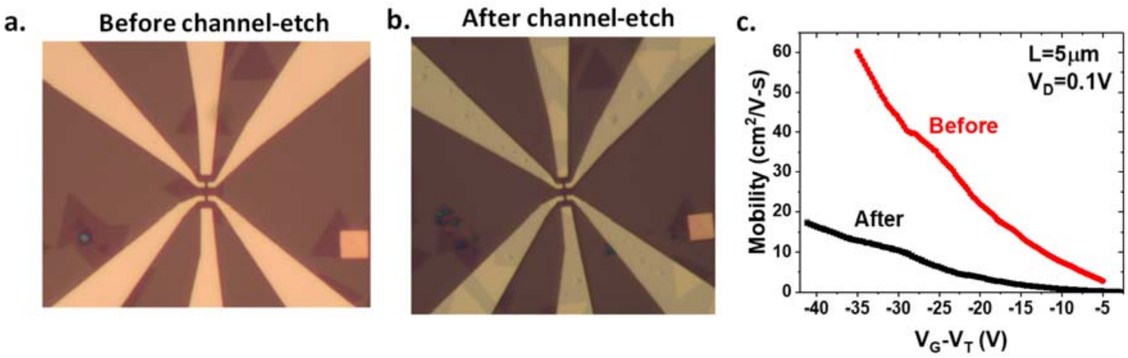

Figure S1. Optical image of a WSe_2 Hall-bar device before (a) and after (b) channel etching. (c) The extracted mobility of the Hall-bar device before and after channel etching and SEM scan.

2. Temperature dependence of channel mobility after channel etching and SEM scan

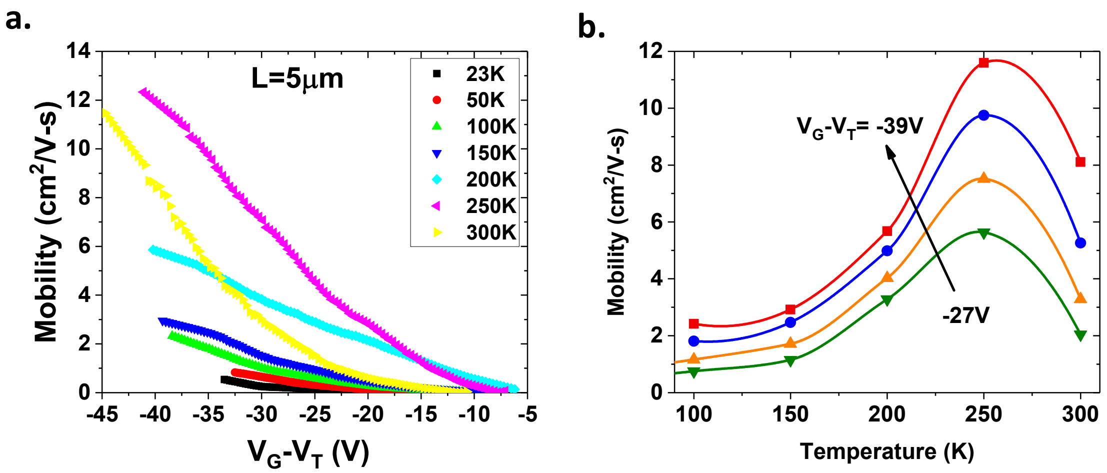

Figure S2. Temperature dependence of channel mobility in a WSe_2 Hall-bar device after channel-cut and SEM scan. (c) Extracted field-effect mobility as a function of gate overdrive, $V_G - V_T$, at various temperatures. (d) Temperature dependence of the field-effect mobility at various gate overdrives.
